# Fractionated vaccination with mRNA COVID‐19 vaccine is safe for patients with polyethylene glycol allergy

**DOI:** 10.1002/clt2.12217

**Published:** 2023-01-21

**Authors:** Charlotte G. Mortz, Henrik F. Kjaer, Torbjørn Kabel Georgsen, Trine H. Rasmussen, Helene M. Rasmussen, Sigurd Broesby‐Olsen, Carsten Bindslev‐Jensen

**Affiliations:** ^1^ Department of Dermatology and Allergy Centre Odense Research Centre for Anaphylaxis (ORCA) Odense University Hospital University of Southern Denmark Odense Denmark

To the editor

1

The safety of COVID‐19 vaccinations in polyethylene glycol (PEG) allergic patients is a challenge as PEG is an excipient in several mRNA vaccines including Comirnaty^®^. The viral vector vaccines containing the excipient polysorbate 80 have been used in some PEG allergic patients. However, not all patients can be handled this way due to possible severe side effects of these vaccines as well as co‐sensitization to polysorbates in PEG allergic patients. We aimed to vaccinate PEG allergic patients with fractionated Comirnaty^®^ vaccine using a four step protocol.

The evaluation for excipient allergy in our patient cohort has previously been described.^1^ Based on further challenges in 2021, 17 patients were diagnosed with PEG allergy (history of an allergic reaction to a PEG containing drug and a positive skin prick test [SPT] or positive challenge to PEG). From December 2021 fractionated Comirnaty^®^ vaccination was offered to patients with PEG allergy. The fractionated Comirnaty^®^ vaccine was administered without premedication using 1/10, 1/5, 1/3 and finally 11/30 of the 0.3 ml dose Comirnaty^®^ with 1 h intervals under anaphylaxis surveillance and observation for at least 2 h after the last dose.

The study was approved by the Danish Data Protection Agency (Journal nr.: 20/63311) and the Ethics Committee (Report nr.: Covid—21/209, nr. 50).

The 17 patients with PEG allergy were divided into PEG allergy with (group 1) and without concomitant polysorbate allergy (group 2). In group 2 (Table 1), 10 of 11 were previously offered a Janssen vaccine^®^. Three accepted and were successfully vaccinated twice.^1^ From December 2021, the remaining seven were offered a fractionated Comirnaty^®^ vaccination. Four were vaccinated without any allergic symptoms, and three abstained due to recent COVID‐19 infection or anxiety for vaccine side effects in general. The last patient, not offered Janssen vaccine^®^ initially, was successfully revaccinated twice with a fractionated Comirnaty^®^ vaccination (referred with reaction to Moviprep laxative and the second Comirnaty^®^ vaccination).

**TABLE 1:** Patients with PEG allergy and unlikely polysorbate allergy

ID	Elicitor WAO grade	Positive SPT (primary evaluation, re‐evaluation)	Positive BaHR (primary evaluation, re‐evaluation)	Specific IgE (PEG 2000/10,000)	Challenge PEG 3350	Primary vaccine offered	Secondary vaccine offered	Result of vaccination
1	Diprospan, Depo‐Medrol steroid injection (PEG 3350) Moviprep laxative (PEG 3350) Escitalopram (PEG 400) WAO 5	PEG 20.000 (0.01%) Generalized urticaria 2 h after SPT ×2	‐	<0.10	Positive	Janssen vaccine® Abstained	Comirnaty® divided in 4 doses Accepted	Successfully vaccinated ×3
57 M								
2	Depo‐Medrol steroid injection (PEG 3350) Panodil Zapp (PEG 6000) WAO 4	PEG 3350, PEG 6000	‐	<0.10	Positive	Janssen vaccine® Abstained	Comirnaty® divided in 4 doses Accepted	Successfully vaccinated ×2
45 F								
4	Diprospan steroid injection (PEG 3350) Panodil Zapp (PEG 6000) WAO 1	PEG 3000, Polysorbate 80 Generalized urticaria during SPT	‐	<0.10	Positive	Janssen vaccine® Abstained	Comirnaty® divided in 4 doses Accepted	Successfully vaccinated ×2
47 F								
13	Movicol laxative (PEG 3350) WAO 5	PEG 6000, PEG 20.000 (10%)	PEG 6000	<0.10	ND	Janssen vaccine® Abstained	Comirnaty® divided in 4 doses Accepted	Successfully vaccinated ×2
34 F								
6	Depo‐Medrol steroid injection (PEG 3350) WAO 2	PEG 20.000 (20%)	PEG 3000, PEG 6000	<0.10	ND	Janssen vaccine® Accepted	Janssen vaccine® Accepted	Successfully vaccinated ×2
46 F								
7	Depo‐Medrol steroid injection (PEG 3350) ×2 WAO 1	‐	Polysorbate 80	<0.10	Positive	Janssen vaccine® Accepted	Janssen vaccine® Accepted	Successfully vaccinated ×2
25 M								
9	Depo‐Medrol steroid injection (PEG 3350) ×3 WAO 2	PEG 20.000 (0.1%)	PEG 3000, PEG 6000, PEG 20.000 (10%), Spikevax®	<0.10	ND	Janssen vaccine® Accepted	Janssen vaccine® Accepted	Successfully vaccinated ×2
34 F								
15	Moviprep laxative (PEG 3350) Comirnaty® (PEG 2000) second dose WAO 2	DMG‐PEG 2000, ALC‐0159 PEG 2000 Both after 2 h	‐	<0.10	ND	Comirnaty® ×2 before referral	Comirnaty® divided in 4 doses Accepted	Successfully re‐vaccinated ×2
59 F								
3	Depo‐Medrol steroid injection (PEG 3350) WAO 5	PEG 6000 (PEG 20.000 not tested at first evaluation)	PEG 3000	< 0.10/0.11	Negative low dose	Janssen vaccine® Abstained	Comirnaty® divided in 4 doses Abstained	Abstained
64 F								
11	Movicol laxative (PEG 3350) WAO 2	‐ Urticaria 1 h after SPT	‐	<0.10	Positive	Janssen vaccine® Abstained	Comirnaty® divided in 4 doses Abstained	Abstained
21 F								
19	Lanzoprazol (PEG 6000) Panodil Zapp (PEG 6000) WAO 5	PEG 6000, PEG 20.000 (10%), Poloxamer 407	PEG 300	<0.10	ND	Janssen vaccine® Abstained	Comirnaty® divided in 4 doses Abstained	Abstained
30 F								

Abbreviation: ND, not done.

Two of those successfully vaccinated with the fractionated vaccine had previously experienced generalized urticaria during or after SPT with PEG. However, the vaccination did not elicitate any symptoms. After vaccination of group 2, the six group 1 patients were offered fractionated vaccination (Table 2). Patients in this group had more positive reactions in SPT and 4/6 had specific IgE to PEG 2000/10,000. Five of the six patients in group 1 accepted a fractionated Comirnaty^®^; four received the vaccination 2–3 times without allergic symptoms and one was re‐vaccinated without reaction. One patient abstained due to COVID‐19 sequelae.

**TABLE 2:** Patients with PEG allergy and polysorbate allergy/possible polysorbate allergy

ID	Elicitor WAO grade	Positive SPT (primary evaluation, re‐evaluation)	Positive BaHR (primary evaluation, re‐evaluation)	Specific IgE (PEG 2000/10,000)	Challenge PEG 3350	Primary vaccine offered	Secondary vaccine offered	Result of vaccination
12–38 M	Movicol laxative (PEG 3350) Infliximab (polysorbate 80) ×2 WAO 5	DMG‐PEG 2000, PEG 3350, PEG 6000, PEG 20,000 (0.01%), Polysorbate 80	‐	0.11/0.63	ND	None	Comirnaty® divided in four doses	Successfully vaccinated ×2
14–31 F	Moxaxole laxative (PEG 3350) Brintellix (PEG 400) WAO 2	PEG 2000, DMG‐PEG 2000, PEG 3350, PEG 6000, PEG 20,000 (0.01%), Poloxamer 407, Polysorbate 20, Polysorbate 80	‐	<0.10	ND	None	Comirnaty® divided in four doses	Successfully vaccinated ×3
16–19 F	Vaxzevria® vaccine (polysorbat 80) Movicol laxative (PEG 3350) WAO 1	PEG 300, PEG 2000, DMG‐PEG 2000, PEG 3000, PEG 6000, PEG 20,000 (0.1%), Poloxamer 407, Polysorbate 20, Polysorbate 80, Spikevax®, Vaxzevria®	Spikevax®, Vaxzevria®, Comirnaty®	<0.10	Positive	None	Comirnaty® divided in four doses (Omalizumab treatment—CSU)	Successfully vaccinated ×2
18–22 F	Primcillin (PEG 6000) Depo‐Medrol steroid injection (PEG 3350) WAO 3	PEG 2000, DMG‐PEG 2000, PEG 3000, PEG 3350, PEG 6000, PEG 20,000 (0.01%), Poloxamer 407, Polysorbat 80, Polysorbat 20	Poloxamer 407, Comirnaty®, Spikevax®	1.24/2.11	ND	None	Comirnaty® divided in four doses	Successfully vaccinated ×2
10–47 F	Depo‐Medrol steroid injection (PEG 3350) WAO 1	DMG‐PEG 2000, ALC‐PEG 2000, PEG 6000, PEG 20,000 (0.1%), Poloxamer 407, Polysorbate 80	DMG‐PEG 2000, ALC‐PEG 2000, Comirnaty®, Spikevax®	0.27/0.53	ND	Comirnaty® ×2 before referral	Comirnaty® divided in four doses	Successfully re‐vaccinated ×1
8–50 F	Diprospan steroid injection (PEG 3350) Movicol laxative (PEG 3350) Vagifem (PEG 6000) WAO 1	PEG 2000, DMG‐PEG 2000, PEG 3000, PEG 3350, PEG 6000, PEG 20,000 (0.01%), Poloxamer 407, Polysorbat 80	PEG 20,000 (10%), Comirnaty®, Spikevax®	22.70/39.10	ND	None	Comirnaty® divided in four doses	Abstained

Anaphylaxis to PEG is rare.^1^ Most reactions described in the literature are elicited by depot‐steroid injections and laxatives.^1^, ^2^ In relation to COVID‐19 mRNA vaccines, PEG was identified as a possible elicitor in case of anaphylaxis, since modified PEG 2000 are used as excipient in mRNA vaccines.^3^, ^4^ Polysorbate 80 is used in viral vector vaccines and a potential cross‐reactivity between PEG and polysorbate has been hypothesized,^2^ due to structural relationship.

During the last years a few case series have described PEG and polysorbate as elicitors in the rare allergic reactions to COVID‐19 vaccines.^5^ A few cases of anaphylaxis to Comirnaty^®^ has been published where PEG allergy was subsequently identified.^3^, ^6^ However, the evidence for PEG or polysorbate 80 being the allergen responsible for anaphylaxis to COVID‐19 vaccines is sparse, and currently alternative mechanisms leading to anaphylaxis are discussed.^7^

Recently, Picard et al.^8^ described two patients with PEG allergy tolerating one step COVID‐19 mRNA vaccine. Furthermore, Brockow et al.^9^ published two patients diagnosed with PEG allergy who were ignorant of the potential risk and tolerated vaccination with Comirnaty^®^. Li et al.^10^ described a PEG allergic patient tolerating mRNA COVID‐19 vaccine in a single step by using pre‐medication. While 0.05 mg of modified PEG 2000 are contained in Comirnaty^®^,^11^ the exact dose in Spikevax^®^ is not stated, but expected to be within the same range. Why these PEG allergic patients tolerated the vaccine with modified PEG 2000 may be explained by the patients individual threshold in relation to the small amount of PEG in the mRNA vaccine, the lower molecular weight of PEG 2000 compared to higher molecular weight PEGs in for example, laxatives and depot‐steroids (PEG 3350), or the conjugation with lipid‐nanoparticles in mRNA vaccines.^11^, ^12^

Titrated vaccination has been described in PEG allergic patients. Faihs et al.^13^ described one patient with a challenge verified PEG allergy receiving fractionated immunization with Spikewax^R^ (five doses given with 30–60 min intervals) without allergic reaction. A single case of oral desensitization with a two‐days, 23 step protocol using serial 10‐fold dilution of PEG 3350 has been published to successfully mRNA COVID‐19 vaccinate a patient.^14^ Picard et al.^8^ also used titrated mRNA vaccination in two of their PEG allergic patients. AlMuhizi et al.^15^ described desensitization to mRNA COVID‐19 vaccine in six patients with a reaction to the first dose of mRNA vaccine with two of the six having immediate reactions during the desensitization.

In conclusion, we could offer a safe COVID‐19 vaccine to all included PEG allergic patients. Three were vaccinated with Janssen vaccine^®^ and 10 with a fractionated Comirnaty^®^ vaccination (4 step protocol) without any allergic reactions or side‐effects. Even patients with urticaria elicited by SPT with PEGs as well as IgE positive to PEG had no reaction during the fractioned vaccination.

## AUTHOR CONTRIBUTIONS

**Charlotte G. Mortz**: Conceptualization (Lead); Data curation (Lead); Formal analysis (Lead); Investigation (Lead); Methodology (Lead); Project administration (Lead); Resources (Lead); Software (Lead); Supervision (Lead); Validation (Lead); Visualization (Lead); Writing – original draft (Lead); Writing – review & editing (Lead). **Henrik F. Kjaer**: Investigation (Equal); Writing – review & editing (Equal). **Torbjorn Kabel Georgsen**: Investigation (Equal); Writing – review & editing (Equal). **Trine H. Rasmussen**: Investigation (Equal); Writing – review & editing (Equal). **Helene M. Rasmussen**: Investigation (Equal); Writing – review & editing (Equal). **Sigurd Broesby‐Olsen**: Writing – review & editing (Equal). **Carsten Bindslev‐Jensen**: Conceptualization (Equal); Investigation (Equal); Writing – review & editing (Equal).

## CONFLICTS OF INTEREST

The authors report no potential conflict of interest.
